# Overcoming Ethical Issues Surrounding the Relationships Between Physicians and Pharmaceutical Companies in Rural Hospitals

**DOI:** 10.7759/cureus.43320

**Published:** 2023-08-11

**Authors:** Ryuichi Ohta, Chiaki Sano

**Affiliations:** 1 Community Care, Unnan City Hospital, Unnan, JPN; 2 Community Medicine Management, Shimane University Faculty of Medicine, Izumo, JPN

**Keywords:** rural hospital, japan, pharmaceutical company, conflict of interests, family medicine

## Abstract

Conflicts of interest (COIs), such as the relationships between pharmaceutical companies and patients and/or their families, can be a significant ethical issue in the field of medicine. The relationship between physicians and pharmaceutical company representatives is just one non-clinical ethical dilemma frequently encountered in medicine. For example, our family medicine department has a COI stemming from its relationship with pharmaceutical companies. We reflected on this COI and discussed a concrete approach from which we implemented evidence-based learning to overcome the challenges presented by this particular COI. This editorial reviews this case and demonstrates an effective approach to addressing COIs.

## Editorial

Relationship between physicians at a rural hospital and pharmaceutical company representatives

Conflicts of interest (COIs), such as the relationships between pharmaceutical companies, patients, and their families, can be a significant ethical issue in medicine [1]. The relationship between physicians and pharmaceutical company representatives is an example of a non-clinical ethical dilemma regularly encountered in medicine and may be related to COIs in clinical practice [1]. Using specific medications manufactured by pharmaceutical companies with which physicians have relationships can breach ethics [2]. When the relationship between a company and a physician is based on the company’s provision of products to the physician, the physician may develop an unprofessional tendency to prescribe medications from the company as opposed to their competitors.

Physicians’ trends of prescriptions in the Department of Family Medicine in Unnan City Hospital were affected by pharmaceutical companies that provide educational lunches to explain their medicines. Although they were not offered money or specific products, they started prescribing specific medicines after participating in the lunch sessions. However, hospital policy did not prohibit such sessions, and other departments engaged in such sessions. Although our department does not allow team members to participate in such sessions, some of the department’s staff participated in sessions hosted by other departments. However, the department members did not notice any changes in the clinical prescription habits of departmental team members who attended the sessions.

Assessment of ethical issues

To assess the ethical issues involved in the relationship between pharmaceutical company representatives and medical staff, it is crucial to have a framework that conceptualizes professionalism. This framework consists of the following: a compassionate, respectful, and collaborative orientation; integrity and accountability; the pursuit of excellence; and fair and ethical stewardship of healthcare resources [3]. In the present case, all of these factors should be considered.

When evaluating this case from a compassionate, respectful, and collaborative perspective, some colleagues tended to prescribe medications based on their relationships with pharmaceutical companies. This tendency violated patient autonomy because the prescribers were affected by the COI and did not respect patient preferences for certain medications [3]. These colleagues chose specific medications without evaluating the available options, contradicting physician professionalism. Patients may experience unknown disadvantages in their care if patient factors are not considered when selecting medications.

When examining integrity and accountability, the colleagues’ behaviors with COIs may impinge on maintaining appropriate relationships with patients and managing COIs involving the public disclosure of any relationships that may affect practitioners’ treatment decisions [3]. In the case presented here, it was initially unknown whether our colleagues recognized that there had been a breach of ethics and medical professionalism. To address this issue, peer colleagues and 360-degree reviews are vital to effectively evaluate and amend employees’ perceptions of their relationships with pharmaceutical companies [4].

In the pursuit of excellence, practitioners depend on information from pharmaceutical companies to update their medical knowledge, which contradicts the ethical responsibilities of medical professionals. Instead, physicians should adjust their learning based on available evidence and applicable guidelines [3] and their professional development based on the curricula and standards of medical societies. Using information from pharmaceutical companies without applying critical thinking or assessing evidence quality can cause a breach of medical ethics. Practitioners not practicing this approach should shift their attitudes toward evidence-based practice.

When assessing the fair and ethical stewardship of healthcare resources, using specific medications can affect the equity of patient access to medications and appropriate medical resources [3]. When physicians prescribe specific medications guided by their relationships with pharmaceutical companies, patients may not get the most affordable medications or may have to pay higher costs to insurance companies or insurers, resulting in diminished equity of access to medications. Therefore, providers’ tendency to prescribe specific medications based on their relationships with pharmaceutical companies breaches medical ethics.

Ethical framework implementation in addressing ethical issues

One framework for addressing ethical issues in medicine is physician resilience, which comprises self-awareness, self-regulation, social awareness, and social regulation [5].

When considering self-awareness, medical professionals should recognize their emotions, triggers, and transferences, which may interfere with appropriate behavior. Additionally, they should understand their limitations in terms of their medical skills and knowledge [5]. In the present case, this was an area wherein my colleagues could be considered unprofessional. The other team members’ reactions to their behavior with COIs included embarrassment and irritation. They were initially unable to control my emotions or talk to their colleagues with COIs professionally because of their feelings toward their colleagues with COIs. However, as a professional, they realized they should not let their emotions skew their attitude toward their colleagues with COIs to not complicate the situation further.

Physicians should temper emotional responses to ethical issues when evaluating self-regulation and understand the complexities of various ethical issues [5]. Dealing with the case presented herein, the team members in the Department of Family Medicine learned how to control their strong emotions toward my colleagues’ unprofessional behaviors. Reflection is a vital method for controlling negative emotions. The team members took time to reflect on their previous unprofessional behaviors and why they felt the way they did. During their time as medical students, they learned about ethical issues. In particular, their ethics teachers strongly opposed the relationship between physicians and pharmaceutical companies. Additionally, the team members had previously been taught about the dangers of decaying relationships, which drove my disdain for relationships with pharmaceutical companies. By reflecting on my time in medical school, they could evaluate and understand the reasons for their strong emotions and gradually alleviate them.

Concerning social awareness, physicians should analyze every situation to identify potential challenges to professionalism and recognize the importance of considering the needs and roles of every individual involved in ethical issues [5]. As discussed previously, multiple potential medical and ethical factors were involved in the present case. Members with COIs have had ethical conflicts in establishing effective relationships with their colleagues because they breached professionalism. To work effectively as part of a team, members with COIs needed to build a better relationship with each individual, free of conflict; however, their breach of medical ethics needed to be addressed to ensure that patients were getting the best care. In the long term, the colleagues’ impartial preferences toward medicines might negatively affect overall patient care; therefore, the team members discussed the need to re-evaluate their relationships with pharmaceutical companies. However, direct accusations against their colleagues with COI were challenging to handle because their emotions and pride must be considered. Additionally, other colleagues may be swayed by pharmaceutical companies in the future. Therefore, social regulations should be considered when implementing evidence-based medicine.

One example of social regulation is the habitual justification for the presence of pharmaceutical companies in hospitals [5]. However, such interventions have been attempted in various hospitals with limited success. In Unnan City Hospital, these attempts were ineffective, and as such, the Department of Family Medicine’s colleagues’ behavior was in line with the present working conditions. In addition, pharmaceutical companies must promote the strengths of their medicines to medical professionals in the form of work functions. Medical professionals’ literacy is essential for rationally assessing the evidence provided by pharmaceutical companies. Pharmaceutical company advertisers should be honest about their products.

Given the ethical breaches in the present case, the department’s team members discussed the status of our relationships with pharmaceutical companies and how to ensure and sustain professionalism. Through this discussion, the Department of Family Medicine decided to host official evidence-based conferences to evaluate the information presented on each medication provided by pharmaceutical companies. During these conferences, the Department of Family Medicine practitioners discussed the effectiveness of the promoted medicines based on evidence from pharmaceutical companies and scientific papers. The decision to administer these medications to our patients was made through evidence-based discussions and adhered to as long as the practitioner belonged to the Department of Family Medicine. Through the implementation of this impartial, evidence-based process, negative feelings in the department faded. Additionally, some team members stated that any practitioner could hold a conference to provide clarity and further evidence for prescribing specific medicines to their patients. Pharmaceutical companies can also demonstrate their competency in explaining the benefits of their medications and collaboratively discuss with physicians to improve their utilization.

Application of the framework for finding common ground and providing community-oriented primary care

To resolve COIs resulting from relationships between physicians and pharmaceutical companies, there is a framework for finding common ground and providing community-oriented primary care (COPC), which can be used to analyze each relationship and find ways to resolve these issues.

Finding common ground in a relationship is beneficial for understanding each stakeholder’s ideas regarding that relationship [6]. In the present case, considering the perspectives of the colleagues and the pharmaceutical company representative could be beneficial in overcoming any negative feelings toward them. In the framework of finding common ground in a patient-centered manner, physicians should seek to understand others based on their individual situations or roles rather than from a physician-centered context. As seen in the present case, we should strive to understand our colleagues’ motivation to learn about medications and the pharmaceutical company’s motivation to convey the uses and benefits of their medications to find common ground. Considering their motivations and perceptions, the team members could control their negative feelings and move forward with concrete interventions to overcome my perceived professional challenges.

In particular, the COPC framework is practical for finding solutions to community issues, including those experienced by hospitals [7]. In the present case, negative feelings were overcome by analyzing the social contexts of the relationships between physician colleagues and pharmaceutical companies. Based on the analysis and discussion of issues in the Department of Family Medicine, new interventions have been established to allow the provision of medical information by pharmaceutical companies. Considering the COPC framework with a cyclic process comprising analyzing issues, revising points, planning interventions, and provision and revision, the present case could guide the family medicine team to provide better professional care.

Need for the interventions and challenges

By navigating the COIs in the relationship between physicians and pharmaceutical companies, the Department of Family Medicine at Unnan City Hospital developed an evidence-based framework for approaching pharmaceutical companies and their medications [8]. As ethical issues can be approached personally and systematically, each team member’s ideas are respected through discussions, and a new format for conferences with pharmaceutical companies has been implemented.

The continuity of these conferences with pharmaceutical companies is unknown, but the long-term effects on departmental team members should be evaluated in the future. Social and environmental conditions can affect medical professionals’ ethical and professional behaviors [8], and there is always the possibility that another COI may occur if the environment of each conference is not controlled. Professional relationships between physicians and pharmaceutical companies should be consistent and based on each professional’s code of ethics.

For the effective continuity of these professional conferences, constant personal and group reflections and discussions are essential to review and revise professional and ethical considerations [9,10]. To date, there is a lack of research on implementing conferences among physicians and pharmaceutical companies to enhance their professional and ethical considerations. Implementing regular reflection and discussion sessions among family medicine departments can change their perceptions and behaviors regarding their daily practices [9,10]. These newly implemented processes should be respected in subsequent conferences and improved based on professional and ethical considerations.

Conclusions

Ethical issues stemming from the relationship between family medicine physicians and pharmaceutical companies can be monitored through evidence-based learning sessions to ensure continuous professional development. Future research should evaluate the effectiveness of this practice to clarify its practicality as an educational approach to medical ethics.
